# Supplementary material for: Complete Mitogenomic Structure and Phylogenetic Implications of the Genus Ostrinia (Lepidoptera: Crambidae)
Source: Insects. 2020 Apr 7;11(4):232. doi: 10.3390/insects11040232 (PMC7240680; doi:10.3390/insects11040232)
Supplement: Supplementary file 1 [file insects-11-00232-s001.pdf]

Table S1. Collection information of adult specimens in this study.

Name	Locality	Sex	Time	Collector
Ostrinia furnacalis	Huocheng, Yili Kazak Autonomous Prefecture, China	Male	08, July 2017	Ping-Ping Qiao
Ostrinia nubilalis	Huocheng, Yili Kazak Autonomous Prefecture, China	Male	10, July 2017	Ping-Ping Qiao
Ostrinia scapulalis	Suihua, Heilongjiang Province, China	Male	08, July 2018	Ping-Ping Qiao & Nan Zhou
Ostrinia zealis	Zhalantun, Inner Mongolia Autonomous Region, China	Male	20, July 2018	Ping-Ping Qiao & Nan Zhou

Table S2. The best partitioning schemes and models for Maximum likelihood (ML) method based on four datasets selected by PartitionFinder.

Dataset	Optimal Partition	Subset Partitions	Best Model
PCG123	partition1	*cox1*_pos1, *cox2*_pos1, *cox3*_pos1, *cytb*_pos1, *atp6*_pos1	TRN+I
	partition2	*atp6*_pos2, *cox2*_pos2, *nad6*_pos2, *nad3*_pos2, *cytb*_pos2, *nad2*_pos2, *nad5*_pos2, *nad4L*_pos2, *nad4*_pos2	HKY+I
	partition3	*nad6*_pos3, *cytb*_pos3, *cox1*_pos3, *nad3*_pos3, *cox3*_pos3, *cox2*_pos3, *nad2*_pos3, *atp6*_pos3	TIM+I+G
	partition4	*atp8*_pos1, *atp8*_pos2, *nad2*_pos1, *nad3*_pos1, *nad6*_pos1	TIM+I
	partition5	*atp8*_pos3, *nad5*_pos3, *nad4L*_pos3, *nad1*_pos3, *nad4*_pos3	TRN+G
	partition6	*nad1*_pos2, *cox3*_pos2, *cox1*_pos2	F81+I
	partition7	*nad4L*_pos1, *nad5*_pos1, *nad1*_pos1, *nad4*_pos1	K81UF+I
PCG12	partition1	*atp6, nad3, nad2, nad6, atp8*	K81UF+G
	partition2	*cox1, cox3, cox2, cytb*	TRN+I
	partition3	*nad4L, nad1, nad4, nad5*	TIM+I
PCG123R	partition1	*cox1*_pos1, *cox2*_pos1, *cox3*_pos1, *cytb*_pos1, *atp6*_pos1	TRN+I
	partition2	*cox1*_pos2, *nad1*_pos2, *atp6*_pos2, *cox2*_pos2, *cox3*_pos2, *nad3*_pos2, *cytb*_pos2	HKY+I
	partition3	*nad6*_pos3, *cytb*_pos3, *cox1*_pos3, *nad3*_pos3, *cox3*_pos3, *cox2*_pos3, *nad2*_pos3, *atp6*_pos3	TIM+I+G
	partition4	*atp8*_pos2, *atp8*_pos1, *nad4L*_pos1, *rrnL, rrnS*	GTR+G
	partition5	*atp8*_pos3, *nad4*_pos3, *nad1*_pos3, *nad5*_pos3, *nad4L*_pos3	TRN+G
	partition6	*nad5*_pos1, *nad1*_pos1, *nad4*_pos1	HKY+I
	partition7	*nad2*_pos1, *nad3*_pos1, *nad6*_pos1	TIM+I
	partition8	*nad6*_pos2, *nad2*_pos2, *nad5*_pos2, *nad4L*_pos2, *nad4*_pos2	HKY+I
PCG12R	partition1	*atp6, nad3, nad2, nad6, atp8*	K81UF+G
	partition2	*cox1, cox3, cox2, cytb*	TRN+I
	partition3	*nad4L, nad1, nad4, nad5*	TIM+I
	partition4	*rrnS, rrnL*	GTR+G

Table S3. The best partitioning schemes and models for Bayesian inference (BI) method based on four datasets selected by PartitionFinder.

Dataset	Optimal partition	Subset partitions	Best model
PCG123	partition1	*atp6*_pos1, *cox3*_pos1, *cox2*_pos1, *cytb*_pos1, *cox1*_pos1	GTR+I
	partition2	*cox1*_pos2, *nad1*_pos2, *atp6*_pos2, *cox2*_pos2, *cox3*_pos2, *cytb*_pos2	HKY+I
	partition3	*nad3*_pos3, *cox1*_pos3, *nad2*_pos3, *atp6*_pos3, *nad6*_pos3, *cox2*_pos3, *cox3*_pos3, *cytb*_pos3	GTR+I+G
	partition4	*atp8*_pos2, *atp8*_pos1, *nad2*_pos1, *nad3*_pos1, *nad6*_pos1	GTR+I

	partition5	*atp8*_pos3, *nad4*_pos3, *nad1*_pos3, *nad5*_pos3, *nad4L*_pos3	GTR+G
	partition6	*nad4L*_pos1, *nad5*_pos1, *nad4*_pos1, *nad1*_pos1	HKY+I
	partition7	*nad6*_pos2, *nad2*_pos2, *nad4L*_pos2, *nad4*_pos2, *nad3*_pos2, *nad5*_pos2	HKY+I
PCG12	partition1	*atp6, cox3, cox2, cytb*	HKY+I
	partition2	*nad2, nad3, nad6, atp8*	HKY+G
	partition3	*cox1*	HKY+I
	partition4	*nad4L, nad1, nad5, nad4*	HKY+I
PCG123R	partition1	*atp6*_pos1, *cox3*_pos1, *cox2*_pos1, *cox1*_pos1, *cytb*_pos1	GTR+I
	partition2	*cox1*_pos2, *nad1*_pos2, *atp6*_pos2, *cox2*_pos2, *cox3*_pos2, *nad3*_pos2, *cytb*_pos2	HKY+I
	partition3	*nad3*_pos3, *cox1*_pos3, *nad2*_pos3, *atp6*_pos3, *nad6*_pos3, *cox2*_pos3, *cox3*_pos3, *cytb*_pos3	GTR+I+G
	partition4	*atp8*_pos2, *atp8*_pos1, *rrnS*, *rrnL*	GTR+G
	partition5	*atp8*_pos3, *nad4*_pos3, *nad1*_pos3, *nad5*_pos3, *nad4L*_pos3	GTR+G
	partition6	*nad4L*_pos1, *nad5*_pos1, *nad1*_pos1, *nad4*_pos1	HKY+I
	partition7	*nad2*_pos1, *nad3*_pos1, *nad6*_pos1	GTR+I
	partition8	*nad6*_pos2, *nad2*_pos2, *nad5*_pos2, *nad4L*_pos2, *nad4*_pos2	HKY+I
PCG12R	partition1	*cox1, atp6, cox3, cox2, cytb*	GTR+I
	partition2	*nad3, nad2, atp8, nad6*	HKY+G
	partition3	*nad4L, nad1, nad5, nad4*	HKY+I
	partition4	*rrnS, rrnL*	GTR+G

Table S4. Mitogenomic organization of *Ostrinia penitalis*.

Gene	Position		Size(bp)	Intergenic nucleotides	Codon		Strand
	From	To			Start	Stop	
nad2	1	575	575			TAA	J
trnW	583	650	68	7			J
trnC	644	705	62	−7			N
trnY	709	770	62	3			N
cox1	781	2316	1536	10	CGA	TAA	J
trnL	2312	2378	67	−5			J
cox2	2379	3080	702		ATT	TAG	J
trnK	3076	3145	70	−5			J
atp8	3211	3372	162	65	ATA	TAA	J
atp6	3366	4040	675	−7	ATG	TAA	J
cox3	4040	4831	792	−1	ATG	TAA	J
trnG	4834	4900	67	2			J
nad3	4901	5254	354		ATT	TAA	J
trnA	5261	5326	66	6			J
trnR	5326	5389	64	−1			J
trnN	5389	5454	66	−1			J
trnS1	5456	5523	68	1			J
trnE	5524	5592	69				J
trnF	5593	5656	64				N
nad5	5641	7377	1737	−16	ATT	TAA	N
trnH	7393	7458	66	15			N
nad4	7458	8798	1341	−1	ATG	TAA	N
nad4L	8805	9098	294	6	ATG	TAA	N
trnT	9107	9173	67	8			J
trnP	9173	9240	68	−1			N
nad6	9241	9777	537		ATT	TAA	J
cytb	9777	10,925	1149	−1	ATG	TAA	J
trnS2	10,925	10,989	65	−1			J
nad1	11,015	11,944	930	25	ATT	TAA	N
trnL1	11,955	12,022	68	10			N
rrnL	12,024	12,612	589	1			N

Table S5. Mitogenomic organization of *Ostrinia palustralis*.

Gene	Position		Size(bp)	Intergenic nucleotides	Codon		Strand
	From	To			Start	Stop	

trnM	1	67	67				J
trnI	68	135	68				J
trnQ	133	201	69	−3			N
nad2	263	1264	1002	61	ATA	TAA	J
trnW	1272	1339	68	7			J
trnC	1332	1399	68	−8			N
trnY	1414	1478	65	14			N
cox1	1487	3017	1531	8	CGA	T	J
trnL2	3018	3084	67				J
cox2	3085	3766	682		ATT	T	J
trnK	3767	3837	71				J
trnD	3840	3907	68	2			J
atp8	3908	4069	162		ATA	TAA	J
atp6	4063	4737	675	−7	ATG	TAA	J
cox3	4737	5528	792	−1	ATG	TAA	J
trnG	5531	5597	67	2			J
nad3	5598	5951	354		ATT	TAA	J
trnA	5954	6020	67	2			J
trnR	6024	6090	67	3			J
trnN	6097	6162	66	6			J
trnS1	6172	6237	66	9			J
trnE	6239	6305	67	1			J
trnF	6304	6369	66	−2			N
nad5	6370	8089	1720		ATT	T	N
trnH	8105	8171	67	15			N
nad4	8171	9511	1341	−1	ATG	TAA	N
nad4L	9520	9813	294	8	ATG	TAA	N
trnT	9822	9888	67	8			J
trnP	9889	9953	65				N
nad6	9956	10,492	537	2	ATT	TAA	J
cytb	10,495	11,640	1146	2	ATA	TAA	J
trnS2	11,640	11,707	68	−1			J
nad1	11,733	12,659	927	25	ATT	TAG	N
trnL1	12,670	12,737	68	10			N
rrnL	12,738	14,069	1332				N
trnV	14,070	14,138	69				N
rrnS	14,139	14,916	778				N
control region	14,917	15,246	330				

Table S6. Mitogenomic organization of *Ostrinia furnacalis*.

Gene	Position		Size(bp)	Intergenic nucleotides	Codon		Strand
	From	To			Start	Stop	
trnM	1	67	67				J
trnI	68	135	68				J
trnQ	136	204	69				N
nad2	266	1267	1002	61	ATA	TAA	J
trnW	1275	1341	67	7			J
trnC	1334	1398	65	−8			N
trnY	1399	1465	67				N
cox1	1474	3004	1531	8	CGA	T	J
trnL2	3005	3071	67				J
cox2	3072	3753	682		ATA	T	J
trnK	3754	3824	71				J
trnD	3824	3892	69	−1			J
atp8	3893	4054	162		ATA	TAA	J
atp6	4048	4722	675	−7	ATG	TAA	J
cox3	4722	5513	792	−1	ATG	TAA	J
trnG	5516	5582	67	2			J

nad3	5583	5936	354		ATT	TAA	J
trnA	5949	6015	67	12			J
trnR	6015	6079	65	−1			J
trnN	6079	6145	67	−1			J
trnS1	6148	6213	66	2			J
trnE	6215	6282	68	1			J
trnF	6281	6349	69	−2			N
nad5	6350	8069	1720		ATC	T	N
trnH	8085	8151	67	15			N
nad4	8151	9491	1341	−1	ATG	TAA	N
nad4L	9499	9792	294	7	ATG	TAA	N
trnT	9802	9868	67	9			J
trnP	9869	9933	65				N
nad6	9936	10,472	537	2	ATT	TAA	J
cytb	10,475	11,620	1146	2	ATA	TAA	J
trnS2	11,620	11,687	68	−1			J
nad1	11,722	12,651	930	34	ATT	TAG	N
trnL1	12,662	12,729	68	10			N
rrnL	12,730	14,070	1341				N
trnV	14,071	14,136	66				N
rrnS	14,137	14,915	779				N
control region	14,916	15,245	330				

Table S7. Mitogenomic organization of *Ostrinia nubilalis*.

Gene	Position		Size(bp)	Intergenic nucleotides	Codon		Strand
	From	To			Start	Stop	
trnM	1	67	67				J
trnI	68	135	68				J
trnQ	136	204	69				N
nad2	267	1268	1002	62	ATA	TAA	J
trnW	1276	1342	67	7			J
trnC	1335	1399	65	−8			N
trnY	1400	1466	67				N
cox1	1475	3005	1531	8	CGA	T	J
trnL2	3006	3072	67				J
cox2	3073	3754	682		ATA	T	J
trnK	3755	3825	71				J
trnD	3825	3893	69	−1			J
atp8	3894	4055	162		ATA	TAA	J
atp6	4049	4723	675	−7	ATG	TAA	J
cox3	4723	5514	792	−1	ATG	TAA	J
trnG	5517	5583	67	2			J
nad3	5584	5937	354		ATT	TAA	J
trnA	5948	6014	67	10			J
trnR	6014	6078	65	−1			J
trnN	6078	6144	67	−1			J
trnS1	6147	6212	66	2			J
trnE	6214	6281	68	1			J
trnF	6280	6348	69	−2			N
nad5	6349	8068	1720		ATT	T	N
trnH	8084	8150	67	15			N
nad4	8150	9490	1341	−1	ATG	TAA	N
nad4L	9498	9791	294	7	ATG	TAA	N
trnT	9801	9867	67	9			J
trnP	9868	9932	65				N
nad6	9935	10,471	537	2	ATT	TAA	J
cytb	10,474	11,619	1146	2	ATA	TAA	J
trnS2	11,619	11,686	68	−1			J

nad1	11,725	12,654	930	38	ATT	TAG	N
trnL1	12,665	12,732	68	10			N
rrnL	12,733	14,071	1339				N
trnV	14,072	14,137	66				N
rrnS	14,138	14,916	779				N
control region	14,917	15,248	332				

Table S8. Mitogenomic organization of *Ostrinia scapulalis*.

Gene	Position		Size(bp)	Intergenic nucleotides	Codon		Strand
	From	To			Start	Stop	
trnM	1	67	67				J
trnI	68	135	68				J
trnQ	136	204	69				N
nad2	266	1267	1002	61	ATA	TAA	J
trnW	1275	1341	67	7			J
trnC	1334	1398	65	−8			N
trnY	1399	1465	67				N
cox1	1474	3004	1531	8	CGA	T	J
trnL2	3005	3071	67				J
cox2	3072	3753	682		ATA	T	J
trnK	3754	3824	71				J
trnD	3824	3892	69	−1			J
atp8	3893	4054	162		ATA	TAA	J
atp6	4048	4722	675	−7	ATG	TAA	J
cox3	4722	5513	792	−1	ATG	TAA	J
trnG	5516	5582	67	2			J
nad3	5583	5936	354		ATT	TAA	J
trnA	5947	6013	67	10			J
trnR	6013	6077	65	−1			J
trnN	6077	6143	67	−1			J
trnS1	6146	6211	66	2			J
trnE	6213	6279	67	1			J
trnF	6278	6346	69	−2			N
nad5	6347	8066	1720		ATT	T	N
trnH	8082	8148	67	15			N
nad4	8148	9488	1341	−1	ATG	TAA	N
nad4L	9496	9789	294	7	ATG	TAA	N
trnT	9799	9865	67	9			J
trnP	9866	9930	65				N
nad6	9933	10,469	537	2	ATT	TAA	J
cytb	10,472	11,617	1146	2	ATA	TAA	J
trnS2	11,617	11,684	68	−1			J
nad1	11,719	12,648	930	34	ATT	TAG	N
trnL1	12,659	12,726	68	10			N
rrnL	12,727	14,065	1339				N
trnV	14,066	14,131	66				N
rrnS	14,132	14,909	778				N
control region	14,910	15,311	402				

Table S9. Mitogenomic organization of *Ostrinia zealis*.

Gene	Position		Size(bp)	Intergenic nucleotides	Codon		Strand
	From	To			Start	Stop	
trnM	1	67	67				J
trnI	68	135	68				J
trnQ	136	204	69				N
nad2	267	1268	1002	62	ATA	TAA	J
trnW	1276	1342	67	7			J

trnC	1335	1399	65	−8			N
trnY	1400	1466	67				N
cox1	1475	3005	1531	8	CGA	T	J
trnL2	3006	3072	67				J
cox2	3073	3754	682		ATA	T	J
trnK	3755	3825	71				J
trnD	3825	3893	69	−1			J
atp8	3894	4055	162		ATA	TAA	J
atp6	4049	4723	675	−7	ATG	TAA	J
cox3	4723	5514	792	−1	ATG	TAA	J
trnG	5517	5583	67	2			J
nad3	5584	5937	354		ATT	TAA	J
trnA	5948	6013	66	10			J
trnR	6013	6077	65	−1			J
trnN	6077	6143	67	−1			J
trnS1	6146	6211	66	2			J
trnE	6213	6278	66	1			J
trnF	6277	6345	69	−2			N
nad5	6346	8065	1720		ATT	T	N
trnH	8081	8147	67	15			N
nad4	8147	9487	1341	−1	ATG	TAA	N
nad4L	9495	9788	294	7	ATG	TAA	N
trnT	9798	9864	67	9			J
trnP	9865	9929	65				N
nad6	9932	10,468	537	2	ATT	TAA	J
cytb	10,471	11,616	1146	2	ATA	TAA	J
trnS2	11,616	11,683	68	−1			J
nad1	11,718	12,647	930	34	ATT	TAG	N
trnL1	12,658	12,725	68	10			N
rrnL	12,726	14,064	1339				N
trnV	14,065	14,130	66				N
rrnS	14,131	14,908	778				N
control region	14,909	15,208	300				
